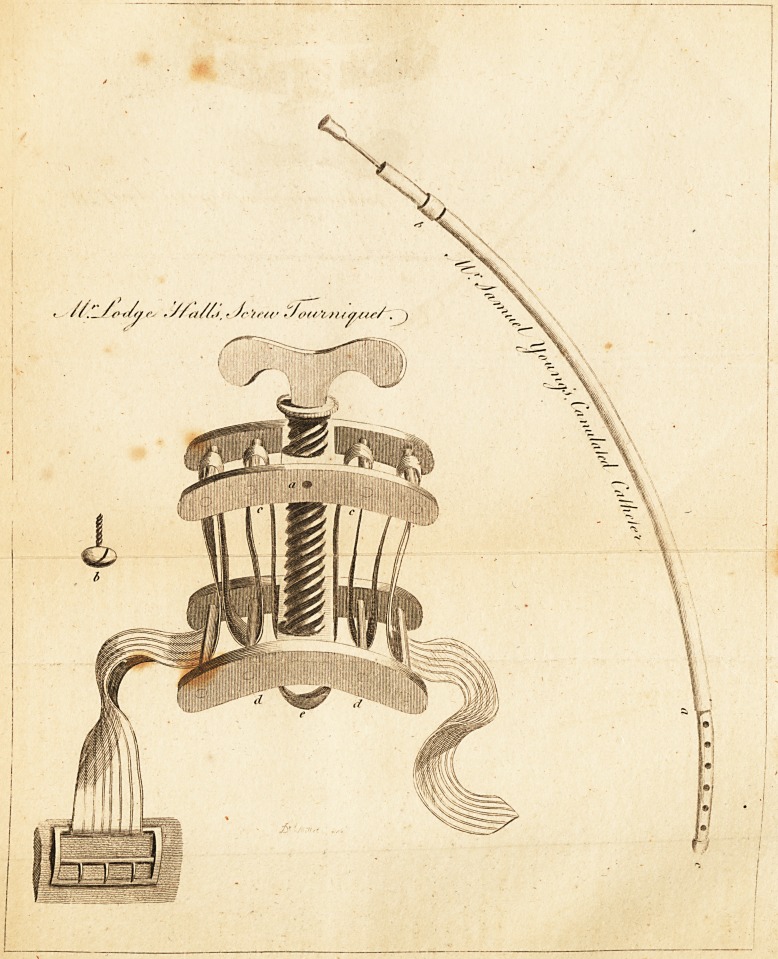# An Attempt to Improve the Screw Tourniquet

**Published:** 1805-12

**Authors:** Lodge Hall

**Affiliations:** Licentiate of the Royal College of Surgeons, in Ireland


### AN ATTEMPT TO IMPROVE THE SCREW TOURNIQUET, *by* MR. LODGE HALL, *Licentiate of the Royal College of Surgeons, in Ireland.*

( With an Engraving. )

NO Instrument since its first introduction has employed the thoughts of Surgeons more than the Tourniquet.

Our predecessors seemed to wish to avail themselves of the knowledge of the pulley and lever; the surgeons of the present day, after having enjoyed the fruits of their labours, as if satiated with the acquirement of ease in stopping the progress of arterial blood through its appropriate tubes, when such stoppage became necessary for the preservation of life, and perhaps anxious, like the rest of the human species, for novelty even at the expence of utility, have endeavoured at simplification; and instruments, little better than the ligature and twisting stick, have been recommended as possessing at least *cheapness* and *ease of manufacture*, superior to the SCREW TOURNIQUET. In my opinion, these ought to be secondary considerations; for, if it can be proved that the lives of many can be rendered more secure by this screw tourniquet than by any other instrument yet invented, it should no more more be laid aside for those less perfect on these accounts than the *matchlock*, because cheaper and less complicated, should be substituted for the *musket*.

It is deemed of national importance to procure the latter on as cheap terms* and *as well executed* as possible. Are such pains then to be taken to procure *proper* instruments of *destruction*? And are they not also to be used in obtaining those of *preservation*?

Every surgeon, of course, should be aware that a garter and any piece of stick can, in the absence of a tourniquet, be used to suppress hæmorrhage; but when a man of science forms one, he should take advantage of what *analogy* and the general laws of mechanism may furnish him with; and after all, if he be successful, the effect will be what is so much desired—*simplicity*.

For what can be more simple than by properly adapting a strap to an instrument with the intervention of a few small rollers, that the blood flowing with such impetus as it is known to do through the largest arteries of the human body, may, in an instant, be retarded by just turning one screw.

Surely, however, necessity may often oblige us to use it; this is more entitled to the appellation of simplicity than that consisting of a piece of tape passed through leather, and in twisting which, it requires not a little caution to prevent its breaking, whilst it *rubs*, *excoriates*, and *pinches* as much as it presses, there being no provision against the effects of friction; this last might rather be termed a *rude*, or an *uncultivated*, than a *simple* instrument.†

Even the Screw Tourniquet, perfect as it is compared to all others, may, I think, be further improved without at all altering the principle laid down by the ingenious inventor Monsieur Petit.

I believe it will be admitted that the male screw of this tourniquet is unwieldy from its great length, particularly when it is, through fear of hæmorrhage, obliged to be left on after any great operation and the patient in bed; nor can it, in its present state be shortened, without at the same time losing considerably in the power of being tightened.

It will also, I believe, be allowed by those in the habit of assisting or performing operations, that it is not always the flow of the blood can be commanded to that exactness with this instrument, (and if not with it, with no other hitherto

* By preventing *extortion*, they are made by *contract*.

† What has been called *the* "Field Tourniquet" is *certainly* more embarrassing, though *probably* not more useful than this.

therto known) that less than three turns of the handle will permit it to pass, and of course as many in the contrary direction stop it.

Both these faults, I think, can be thus *simply* corrected. Let two rollers, such as are already to this instrument, be added, one on each side of the *upper brass plate*, there will then be an equal number (four) above and below. *Two* of those in the *lower brass plate* have always been *totally unnecessary*, as it has been hitherto used. To be convinced of this, let the reader open Mr. B. Bell's System of Surgery on the subject of the ligature of arteries, (it is in the 1st vol. of the third edition, which I have) and he will there see a plate demonstrating this clearly. I have also a tourniquet now before me, I obtained a short time ago from one of the principal makers in London, in which, though the strap is passed over these, there is *not the least necessity* for them, as each side of the tape should be drawn up independently of the *other* side, the part immediately under the rivet of the screw being the fixed point;* and it is almost unnecessary for me to remark the absurdity of providing against *friction*, where *motion* is not intended.

Now I make use of these formerly supernumerary rollers *below*, and the two I have added *above*, as shewn in the drawing better than it is *possible* to describe it.

The power of the SCREW TOURNIQUET is in this manner so increased, that *one* turn of the handle draws up *exactly* as much tape as *two* did before, and the screw may therefore be reduced to *one half* the former allotted length, and still the strap will be shortened from any given dimension, in the same proportion as formerly, with (as already noticed) the advantage of being able to tighten or relax it with *precisely half* the number of turns of the screw.

There is also added, what has been so often attempted unsuccess-

---

* And keeping this in remembrance, the buckle should be always left at some distance from, and not, as is usual, brought close up to the roller; perhaps the best rule would be, to plan the buckle diametrically opposite where the tourniquet is placed; this is by no means so trifling a circumstance as might be imagined, as by neglecting it the difficulty of turning the screw is rendered much greater; for, the buckle preventing the strap from passing up on *one* side, it of course must pass over all the rollers of *both* sides were there an hundred.

Of this also any person may convince himself by applying the tourniquet, alternately with the buckle close up to and at a distance from the roller.

unsuccessfully, viz. a *simple unembarrassing* method of stopping the screw from being relaxed, where it is necessary to allow the tourniquet to remain fixed on a limb, in order to save the life of a patient, till proper measures can be taken to secure any large artery, or even to enable a surgeon to perform an operation alone,* should he be so circumstanced as to have no alternative but the death of one given in his charge—Thus is it effected: A hole is made in the thickest and most central part of the *upper* brass plate, at one of the *sides* where there are no rollers; this admits a small horizontal screw to oppose its point firmly against any part of the large perpendicular one, in such manner as to render it immoveable by any thing accidentally interfering with the handle; and this, I believe, is all that as necessary.†

The alterations here proposed, it is evident, do not in any way affect the usual mode of application of the Screw Tourniquet, nor is any difficulty created but that of passing the strap over *two* more rollers; and this, I hope, is compensated by the advantages arising therefrom.

Any trouble I may have had in attempting this improvement I shall deem amply repaid, if it meet the approbation of those who, in the service of their country or the community, are placed in situations requiring their assistance.

### EXPLANATION OF THE DRAWING.

a. A hole for receiving the screw b. to stop the handle from turning, by opposing its point firmly against the large perpendicular screw connected with it.

c. c. Additional rollers.—d. d. Rollers which were *useless* in the tourniquet as hitherto constructed; their use here is evident.

e. The fixed point from whence the tape (when the tourniquet is applied) should not move.

The remaining parts need no references.

### *Particulars to be attended to by Instrument Makers.*

The rollers should be moderately thick, and should not be polished, but rather made artificially rough, that the tape may catch on and make them turn; as every roller which does not thus turn, has the same effect in retarding motion as a drag placed on the wheel of a carriage.

The rollers should be at such a distance from each other, and from the middle pillars of the plates, that a double of *strong, thick* tape may pass easily

* Without other surgical aid.

† The merit of this part of the improvement (or alteration) belongs to the ingenious artist, who made the instrument from which the drawing (where it may be seen) was taken, Mr. Byrom, North Main Street, Cork.

easily between them; and the *upper* ones should be placed in the intervals of the *lower* ones, thus:

A piece of thick smooth leather should be placed under the whole inferior part retained, by passing the strap through slits cut in it.

## Figures and Tables

**Figure f1:**
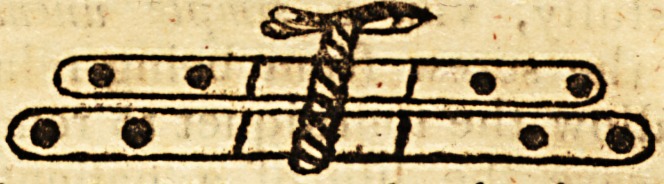


**Figure f2:**